# Metabolomic responses to high-intensity interval exercise in equine skeletal muscle: effects of rest interval duration

**DOI:** 10.1242/jeb.246896

**Published:** 2024-02-16

**Authors:** Kenya Takahashi, Kazutaka Mukai, Yuji Takahashi, Yusaku Ebisuda, Hideo Hatta, Yu Kitaoka

**Affiliations:** ^1^ Department of Sports Sciences, The University of Tokyo, Tokyo 153-8902, Japan; ^2^ Sports Science Division, Equine Research Institute, Japan Racing Association, Tochigi 329-0412, Japan; ^3^ Department of Human Sciences, Kanagawa University, Kanagawa 221-8686, Japan

**Keywords:** Metabolomics, High-intensity interval exercise, Thoroughbred, Skeletal muscle

## Abstract

High-intensity interval training has attracted considerable attention as a time-efficient strategy for inducing physiological adaptations, but the underlying mechanisms have yet to be elucidated. By using metabolomics techniques, we investigated changes in the metabolic network responses in Thoroughbred horses to high-intensity interval exercise performed with two distinct (15 min or 2 min) rest intervals. The peak plasma lactate level was higher during high-intensity exercise with a 2 min rest duration than that with a 15 min rest duration (24.5±6.8 versus 13.3±2.7 mmol l^−1^). The arterial oxygen saturation was lower at the end of all exercise sessions with a 2 min rest duration than that with a 15 min rest duration. Metabolomic analysis of skeletal muscle revealed marked changes in metabolite concentrations in the first and third bouts of the 15 min rest interval conditions. In contrast, there were no metabolite concentrations or pathways that significantly changed during the third bout of exercise performed with a 2 min rest interval. Our findings suggest that the activity of each energy production system is not necessarily reflected by apparent changes in metabolite concentrations, potentially due in part to a better match between metabolite flux into and out of the pathway and cycle, as well as between metabolite production and disposal. This study provides evidence that changes in metabolite concentrations vary greatly depending on the number of repetitions and the length of rest periods between exercises, even if the exercises themselves are identical.

## Teaser

**Summary:** Metabolic responses of Thoroughbred horses to the same high-intensity exercise differ not only with repetition but also with the length of the rest period between exercise bouts.

## INTRODUCTION

High-intensity interval exercise induces marked elevations in adenosine triphosphate (ATP) turnover and provokes considerable metabolic perturbations, which are thought to explain the temporal efficiency of high-intensity interval training for promoting skeletal muscle remodeling and enhancing exercise performance (MacInnis et al., 2017). In addition to exercise intensity, rest interval duration during interval exercise may play a key role in facilitating physiological adaptations. In humans, some studies have reported that rest interval duration does not affect muscle or performance adaptations (Edge et al., 2013; McGinley and Bishop, 2017), while other studies have reported that improvements of exercise performance after a training period were influenced by rest duration-dependent metabolic fluctuations (Fiorenza et al., 2018; Mohr et al., 2007). This inconsistency is possibly due to the magnitude of metabolic stress caused by the difference in exercise intensity and/or duration of each bout and total number of bouts, as well as rest interval duration. Therefore, to elucidate the role of the rest period itself in high-intensity interval exercise, it is not sufficient to examine the concentration of metabolites in skeletal muscle simply before and after exercise, but it is necessary to investigate the time course in detail, including during the rest intervals.

Thoroughbred horses possess extraordinary physiological properties, such as high maximal oxygen uptake (Jones et al., 1989) and muscle glycogen content (Lindholm et al., 1974), allowing them to exhibit superior performance in high-intensity exercise. Their remarkable athletic characteristics may provide profound insights into exercise metabolism, and as model animals they also have the notable advantage of being able to have their muscle sampled repeatedly in the short periods between exercises. Considering recent evidence that various metabolites act as signaling molecules (Chow et al., 2022; Kitaoka et al., 2016; Martinez-Reyes and Chandel, 2020), we used unbiased metabolite profiling of skeletal muscle of Thoroughbred horses performing high-intensity interval exercise, obtained before and after the first and third exercise sessions, with the aim of identifying candidate metabolites for training adaptations. To better understand the role of rest duration in muscle energy metabolism, we compared exercise protocols with 15 min and 2 min recovery periods.

## MATERIALS AND METHODS

### Animals and ethical approval

Eight Thoroughbred horses (5 geldings and 3 mares; age 3–7 years old; body mass 515±68 kg; maximal oxygen uptake 185±14 ml kg^−1^ min^−1^) were used in this study. The experimental protocols for the study were reviewed and approved by the Animal Welfare and Ethics Committee of the Japan Racing Association (JRA) Equine Research Institute (Approval number: 20-11, 20-12). All incisions for catheter placement and muscle biopsies were performed under local anesthesia using lidocaine. All efforts were made to minimize animal discomfort.

### Preliminary incremental exercise test

Incremental exercise tests were conducted 1 week before the first session of high-intensity interval exercise. The procedures for the incremental exercise test, including oxygen consumption measurements, have been described previously (Kitaoka et al., 2011; Mukai et al., 2017). Briefly, the horses warmed up by trotting at 4 m s^−1^ for 3 min; then, an open-flow mask was fitted to each horse, and the horses began exercising up a 6% incline for 2 min each at 1.7, 4, 6, 8, 10, 12 and 13 m s^−1^ until the horses could not maintain their positions at the front of the treadmill with human encouragement.

Horses wore an open-flow mask on the treadmill through which a rheostat-controlled blower drew air. Air flowed through 25 cm diameter tubing and across a pneumotachograph (LF-150B, Vise Medical, Chiba, Japan) connected to a differential pressure transducer (TF-5, Vise Medical). O_2_ and CO_2_ concentrations were measured with an O_2_ and CO_2_ analyzer (MG-360, Vise Medical), and gas analyzer outputs for the final 30 s of each step were also recorded on personal computers using commercial hardware and software (DI-720 and Windaq Pro+, DATAQ, Akron, OH, USA) with sampling at 200 Hz.

### High-intensity interval exercise

**Fig. 1.**
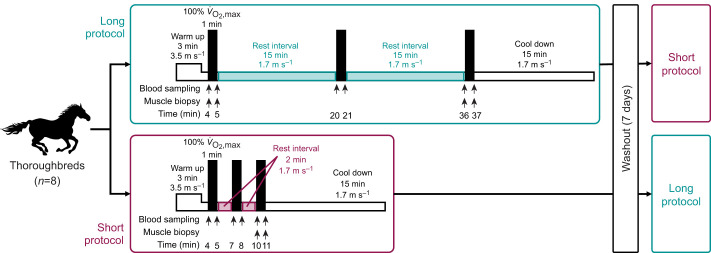
**Schematic diagram of the exercise protocol.** High-intensity interval exercise using the long and short protocols in a randomized crossover design.

High-intensity interval exercise was carried out following the methods of a previous study with some modifications (Eto et al., 2004) (Fig. 1). In a randomized crossover design, eight Thoroughbred horses performed high-intensity interval exercise on a treadmill with a 7 day washout period between trials. After warm-up exercise at 3.5 m s^−1^ for 3 min, followed by 1.7 m s^−1^ for 1 min, horses performed three 1 min bouts of high-intensity exercise at the speed eliciting their maximal oxygen uptake, separated by 15 min or 2 min of walking at 1.7 m s^−1^ (Fig. 1). Exercise protocols with 15 min and 2 min rest intervals were defined as long and short protocols, respectively. Heart rate was recorded using a commercial heart rate monitor (S810, Polar, Kempele, Finland), and mean heart rate was calculated for the final 30 s of each step.

### Blood and muscle sampling

Before a horse was led onto the treadmill, an 18-gauge catheter (Surflow, Terumo, Tokyo, Japan) was placed in its carotid artery, and an 8-F introducer (MO95H-8, Baxter International, Deerfield, IL, USA) was placed in its jugular vein. A Swan-Ganz catheter (SP5107U, Becton, Dickinson and Company, Franklin Lakes, NJ, USA) was passed via the jugular vein so that its tip was positioned in the pulmonary artery. Arterial blood samples were drawn from the 18-gauge carotid catheter at timed intervals into heparinized syringes for the final 10 s of each exercise and were stored on ice until analysis immediately following the experiment. Blood samples were analyzed with a blood gas analyzer (ABL800 FLEX, Radiometer, Copenhagen, Denmark), and O_2_ saturation (*S*_O_2__) and concentration were measured with a hemoximeter (ABL80 FLEX-CO-OX, Radiometer). Following measurement of blood gases and oximetry, the blood was sampled for plasma lactate concentration with a lactate analyzer (Biosen S-Line, EKF-diagnostic GmbH, Barleben, Germany) after being centrifuged at 1740 ***g*** for 10 min. The Swan-Ganz catheter in the pulmonary artery was connected to a cardiac output computer (COM-2, Baxter International) so that its thermistor registered pulmonary arterial temperature, which was recorded at each blood sampling and used to correct the blood gas measurements.

The muscle biopsy sampling site was set at one-third of the distance from the coxal tuber on an imaginary line drawn from the coxal tuber to the root of the tail. After shaving and aseptic preparation, the skin was locally anesthetized by subcutaneous injection of 0.5 ml 2% lidocaine (Sandoz K. K., Tokyo, Japan). Given that sampling depth has been shown to affect muscle fiber-type composition (Rivero et al., 1993), but high reproducibility has been demonstrated when samples are taken from the same depth within a 5 cm radius (Wood et al., 1988), muscle samples were obtained from the same area at a 5 cm depth of the middle gluteal muscle using a 13-gauge×3.9 cm coaxial introducer needle for accurately defining depth and a 14-gauge×9 cm biopsy needle (SuperCore Biopsy instrument, Argon Medical Devices, Plano, TX, USA) immediately before and after the first and third bouts of the long protocol and immediately before and after the third bout of the short protocol (Fig. 1). According to the previous observations by our group examining the effects of multiple muscle biopsies (Kawai et al., 2013), each sampling point was approximately 2 cm apart. All muscle samples were immediately frozen in liquid nitrogen and stored at −80°C until analysis.

### Metabolomics of skeletal muscle

Metabolomic measurements were carried out at Human Metabolome Technologies, Inc. (Tsuruoka, Japan). Frozen muscle specimens (approximately 40 mg) were added to 750 µl of 50% acetonitrile/Milli-Q water containing internal standards (H3304-1002; Human Metabolome Technologies, Inc.) at 0°C to inactivate enzymes. The muscle was homogenized (3 times at 3500 rpm for 120 s) using a tissue homogenizer (Micro Smash MS100R; Tomy Digital Biology Co., Ltd, Tokyo, Japan), and the homogenate was centrifuged (2300 ***g*** at 4°C for 5 min). Subsequently, 400 µl of the upper aqueous layer was centrifugally filtered through a Millipore 5 kDa cutoff filter (9100 ***g*** at 4°C for 120 min) to remove proteins. The filtrate was centrifugally concentrated and resuspended in 50 µl of Milli-Q water for capillary electrophoresis–mass spectrometry (CE-MS) analysis. The metabolites were analyzed using CE-time of flight (TOF) MS (Agilent CE-TOFMS system) and CE-QqQMS (Agilent CE and 6460 Triple Quad LC/MS systems; Agilent Technologies, Santa Clara, CA, USA). Cationic and anionic metabolites were analyzed using a fused-silica capillary (50 µm i.d.×80 cm) with cation buffer solution (p/n: H3301-1001; Human Metabolome Technologies, Inc.) and anionic buffer solution (p/n: H3302-1023; Human Metabolome Technologies, Inc.), respectively, as the electrolyte. CE-TOFMS and CE-QqQMS data were analyzed using automatic integration software (MasterHand v.2.17.1.11, Keio University, Japan) and MassHunter (Agilent Technologies), respectively.

### Statistical analysis

All data were analyzed using MetaboAnalyst software (v.5.0, https://www.metaboanalyst.ca/) and GraphPad Prism software (v.10.1.1, Macintosh, GraphPad Software, La Jolla, CA, USA). Two-way analysis of variance (ANOVA) (bout×protocol) was used for analysis of physiological parameters and the sum of metabolites [glycolytic and tricarboxylic acid (TCA) intermediates]. When an interaction was found to be significant, Tukey's multiple comparison test was performed to determine the differences among the groups. For metabolomic data across the six groups, one-way ANOVA was used. Using the calculated *P*-value of the one-way ANOVA, the adjusted *P*-value [false discovery rate (FDR)] was calculated according to the method of Benjamini and Hochberg. After the FDR cutoff, Tukey's HSD test was used to determine the differences among groups. Metabolite concentrations measured pre- and post-exercise of each exercise bout were compared using a paired *t*-test, followed by the method of Benjamini and Hochberg (FDR cutoff). Metabolite set enrichment analysis and pathway impact analysis were performed using the Kyoto Encyclopedia of Genes and Genomes (KEGG) database. Unless otherwise noted, all data are expressed as means±s.d. or means with individual values. Statistical significance was defined as *P*<0.05 and FDR<0.05 (cutoff).

## RESULTS

### Heart rate and blood measurements

#### Heart rate

**Fig. 2.**
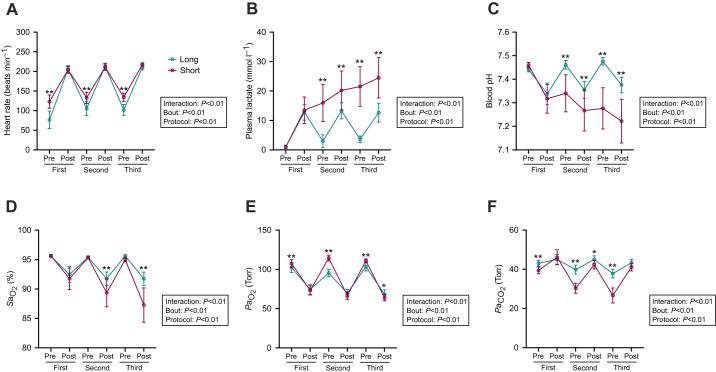
**Heart rate and blood measurements during high-intensity interval exercise.** Heart rate (A), plasma lactate (B), blood pH (C), arterial oxygen saturation level (*S*a_O_2__, D), arterial oxygen partial pressure (*P*a_O_2__, E) and arterial carbon dioxide partial pressure (*P*a_CO_2__, F) during high-intensity interval exercise. Two-way analysis of variance (ANOVA) followed by Tukey's multiple comparison test was performed. Data are expressed as means±s.d. (*n*=8). Asterisks indicate significant differences between the two protocols at the same time point or in the same bout (**P<0.01, *P<0.05).

Although the heart rate of each horse before each exercise was significantly higher in the short protocol than in the long protocol, the post-exercise heart rate did not significantly differ between horses undergoing the two protocols (Fig. 2A).

#### Plasma lactate

While plasma lactate levels repeatedly increased and decreased throughout the long protocol, progressive increases in plasma lactate levels toward the end of exercise were noted in the horses completing the short protocol, resulting in a significantly higher plasma lactate level in these horses than in those completing the long protocol at the end of the third bout (Fig. 2B).

#### Blood pH

The blood pH level decreased in the horses after each exercise bout but returned to baseline levels before the second and third bouts in the long protocol, whereas a gradual decrease in blood pH was observed in the horses during the short protocol, creating significant differences at the end of the third bout between the two protocols (Fig. 2C).

#### Arterial oxygen saturation

Although the reduction in the *S*a_O_2__ of the horses after the first bout was comparable between the long and short protocols, the *S*a_O_2__ of the horses after the second and third bouts was significantly lower during the completion of the short protocol than during the long protocol (Fig. 2D).

#### Arterial oxygen and carbon dioxide partial pressure

The arterial oxygen partial pressure (*P*a_O_2__) in the horses before each exercise was lower during completion of the long protocol than during completion of the short protocol (Fig. 2E). A significantly lower *P*a_O_2__ was noted after the third bout of the short protocol versus the long protocol. The arterial carbon dioxide partial pressure (*P*a_CO_2__) in the horses before each exercise and after the second bout was significantly higher during completion of the long protocol than during the short protocol (Fig. 2F).

### Metabolomics

**Fig. 3.**
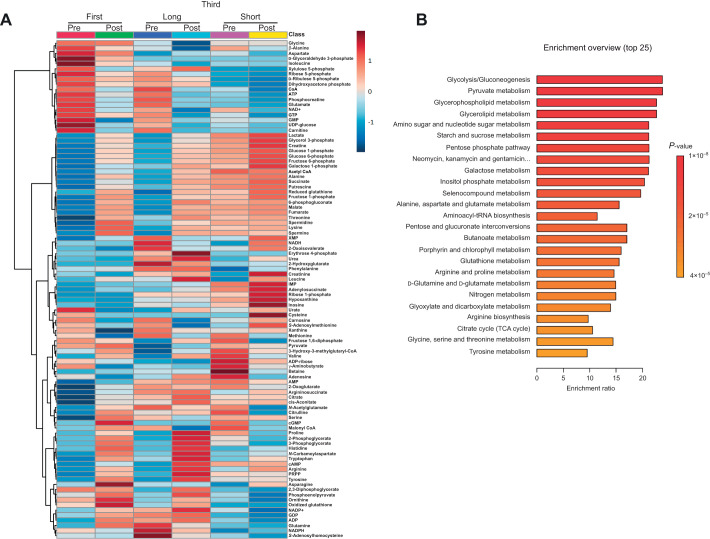
**Overview of metabolomics data.** (A) Hierarchical clustering analysis of detected metabolites, measured pre- and post-exercise from the first and third bouts of the long protocol and the third bout of the short protocol. (B) Metabolite set enrichment analysis was performed using the Kyoto Encyclopedia of Genes and Genomes (KEGG) database.

Among the 116 targeted metabolites, 99 metabolites were detected in the horses in this study (Table S1). Fig. 3A shows a heatmap of the hierarchical cluster analysis of the detected metabolites. Metabolite enrichment analysis revealed that glycolysis/gluconeogenesis and pyruvate metabolism were the top two most significantly altered metabolite sets (Fig. 3B).

#### Identification of significant features via metabolomics

**Fig. 4.**
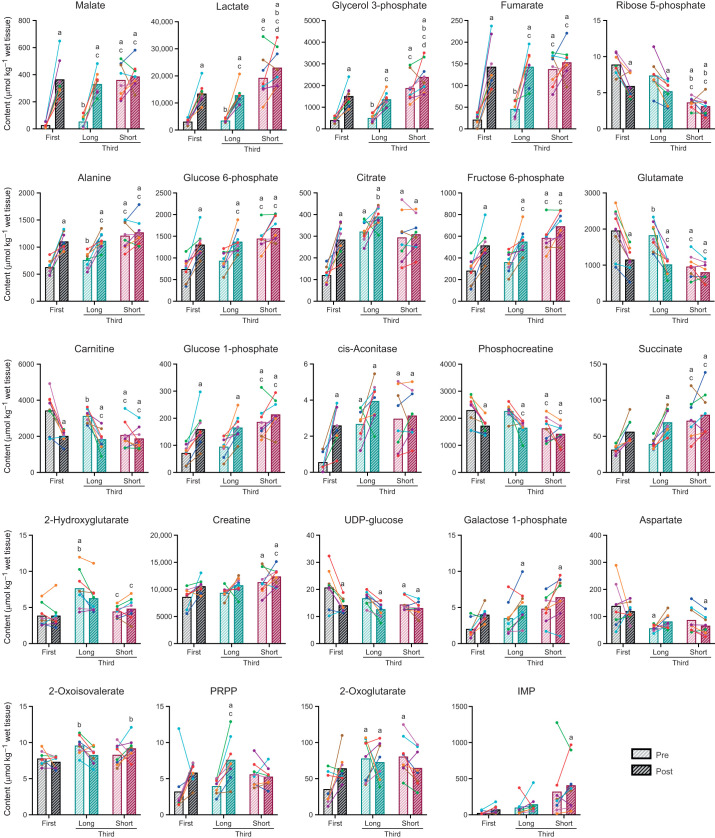
**Significant features of metabolomics.** Pre- and post-first and third exercise bout levels of malate, lactate, glycerol 3-phosphate, fumarate, ribose 5-phosphate, alanine, glucose 6-posphate, citrate, fructose 6-phosphate, glutamine, carnitine, glucose 1-phosphate, cis-aconitase, phosphocreatine, succinate, 2-hydroxyglutarate, creatine, UDP-glucose, galactose 1-phosphate, aspartate, 2-oxoisovalerate, phosphoribosyl pyrophosphate (PRPP), 2-oxoglutarate and inosine monophosphate (IMP) in the long and short protocols. Significant features were detected using the *P*-value of one-way ANOVA, followed by the method of Benjamini and Hochberg [false discovery rate (FDR)<0.05]. After the FDR cutoff, Tukey's HSD test was used to locate the differences among the groups. Data are expressed as means with individual values (*n*=8). a: significant difference from pre-first bout (*P*<0.05). b: significant difference from post-first bout (*P*<0.05). c: significant difference from the pre-third bout in the long protocol (*P*<0.05). d: significant difference from post-third bout in the short protocol (*P*<0.05).

As a result of one-way ANOVA followed by the method of Benjamini and Hochberg, 24 metabolites were identified as significant features (Fig. 4), which primarily included glycolytic and TCA intermediates.

#### Sum of glycolytic and TCA intermediates

**Fig. 5.**
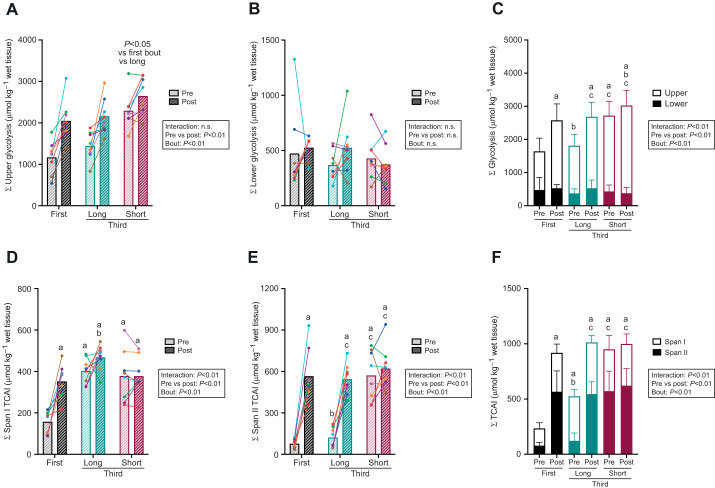
**Sum of the glycolytic and tricarboxylic acid cycle intermediates content.** Pre- and post-first and third exercise bout levels of metabolites involved in upper glycolysis (A) and lower glycolysis (B), total glycolysis (C), span I tricarboxylic acid cycle intermediates (TCAI; D), span II TCAI (E), and total TCAI (F) in the long and short protocols. Two-way analysis of variance (ANOVA) (bout×protocol), followed by Tukey's multiple comparison test, was performed to locate the differences among the groups. Data are expressed as means±s.d. or means with individual values (*n*=8). a: significant difference from pre-first bout (*P*<0.05). b: significant difference from post-first bout (*P*<0.05). c: significant difference from the pre-third bout in the long protocol (*P*<0.05). n.s. not significant.

To better understand glycolysis and TCA metabolism, two major metabolic pathways or cycles related to ATP provision, we calculated the total amount of glycolytic and TCA intermediates according to known divisions. A main effect of exercise bout and protocol was observed in the sum of upper glycolysis (Σ upper glycolysis; from glucose 6-phosphate to fructose 1,6-disphosphate) (Fig. 5A), whereas no interaction or main effect in Σ lower glycolysis (from 2,3-diphosphoglycerate to pyruvate) was noted (Fig. 5B). The total amount of glycolytic intermediates (Σ upper glycolysis+Σ lower glycolysis) increased in the first and third bouts of the long protocol but did not change in the third bout of the short protocol (Fig. 5C). In the first bout, a significant increase was observed in the levels of metabolites involved in the sum of span I TCA intermediates (Σ span I TCAI; from citrate to 2-oxoglutarate). Although no significant increase in the level of metabolites involved in Σ span I TCAI was found in the third bout regardless of the protocol, the Σ span I TCAI level was higher in the third bout of the long protocol than in the first bout (Fig. 5D). There was a significant increase in the level of metabolites involved in Σ span II TCAI (from succinate to oxaloacetate) in the first and third bouts of the long protocol but not in the third bout of the short protocol. No significant difference in the level of metabolites involved in Σ span II TCAI was found between the first bout pre-exercise and the third bout pre-exercise in the long protocol. Additionally, the Σ span II TCAI before and after the third bout in the short protocol was comparable to that after the first and third bouts in the long protocol (Fig. 5E). Similarly, the level of metabolites involved in Σ total TCAI (Σ span I+Σ span II) increased after the first and third bouts in the long protocol but not after the third bout in the short protocol. Moreover, the Σ TCAI level before the third bout in the long protocol was lower than that after the first bout but was higher than that before the first bout (Fig. 5F).

#### Comparison between before and after each exercise bout

**Fig. 6.**
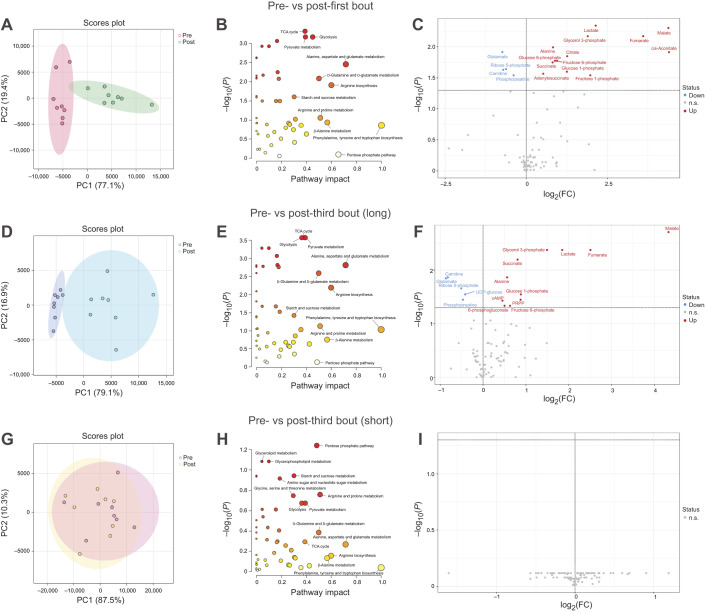
**Comparison between pre- and post-first and pre- and post-third exercise bouts.** Principal component analysis (PCA) (A,D,G), pathway impact analysis (B,E,H) and volcano plot (C,F,I) of pre- and post-first (A–C) and third (D–I) bouts in the long (D–F) and short (G–I) protocols. Principal component (PC)1 and PC2 were plotted with their 95% confidence intervals. Pathway impact analysis was performed using the Kyoto Encyclopedia of Genes and Genomes (KEGG) database; the calculated *P*-value was adjusted by the method of Benjamini and Hochberg (FDR). Metabolite concentrations between pre- and post-exercise bouts were compared using a paired *t*-test, followed by the method of Benjamini and Hochberg (FDR<0.05).

Principal component analysis (PCA) separated the pre- and post-exercise results of the first bout (Fig. 6A), third bout in the long protocol (Fig. 6D) and short protocol (Fig. 6G). Pathway impact analysis revealed that 17 pathways were significantly changed in the first bout (Fig. 6B) and third bout exercise of the long protocol (Fig. 6E), with the TCA cycle, glycolysis and pyruvate metabolism being the top three most significantly altered under both conditions. A volcano plot demonstrated that levels of 13 metabolites increased and the levels of four metabolites decreased in the first bout (Fig. 6C), whereas the levels of 11 metabolites increased and the levels of five metabolites decreased in the third bout of the long protocol (Fig. 6F). Unlike those of the first and third bouts of exercise in the long protocol, the PCA results of the pre- and post-third bouts of exercise in the short protocol overlapped. There were no pathways that were significantly changed in the third bout of the short protocol (Fig. 6H). Additionally, no significant changes were found in the detected metabolites (Fig. 6I).

#### Comparison between before and after all exercise sessions

**Fig. 7.**
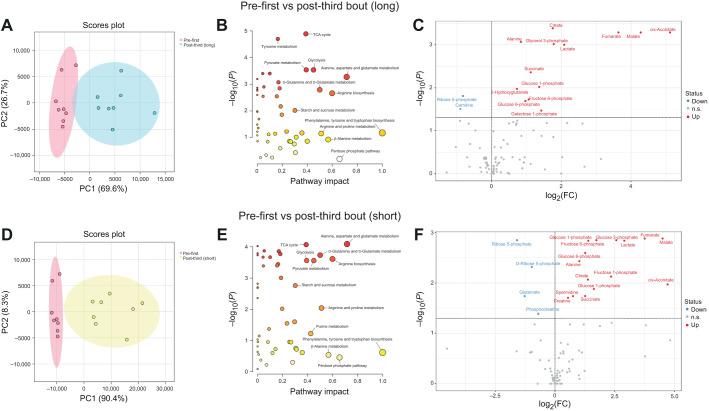
**Comparison between before and after the long and short protocols.** PCA (A,D), pathway impact analysis (B,E) and volcano plot (C,F) before and after the long and short protocols. PC1 and PC2 were plotted with their 95% confidence intervals. Pathway impact analysis was performed using the Kyoto Encyclopedia of Genes and Genomes (KEGG) database. Metabolite concentrations between pre- and post-exercise bouts were compared using paired *t*-tests, followed by the method of Benjamini and Hochberg (FDR<0.05).

Metabolomic profiling results of pre-first bout and post-third bout in the long and short protocols were separated by PCA (Fig. 7A,D). Pathway impact analysis revealed that the TCA cycle, tyrosine metabolism, pyruvate metabolism and glycolysis were the top four most significantly altered pathways after the long protocol (Fig. 7B). Although these pathways were also altered after the short protocol, alanine, aspartate and glutamate metabolism pathways were changed most significantly (Fig. 7E). The volcano plot shows that the levels of 13 metabolites increased and the levels of two metabolites decreased following the long protocol (Fig. 7C), while the levels of 15 metabolites increased and the levels of four metabolites decreased following the short protocol (Fig. 7F).

#### Comparison between the long and short protocols

At the onset of the third bout of exercise in the long and short protocols, the PCA results showed that the individual plots of the principal components did not overlap; however, the 95% confidence interval of the results of the short protocol overlayed that of the long protocol (Fig. S1A). An overlay of the principal components, including the 95% confidence interval, was observed at the end of the long and short protocols (Fig. S1D). Pathway impact analysis showed that the expression levels of 15 pathways were significantly different between the long and short protocols before the start of the third bout (Fig. S1B), but no pathway differed significantly between the post-third bout in the long protocol and that in the short protocol (Fig. S1E). Before the third bout, the levels of nine metabolites were higher and the levels of six metabolites were lower in the short protocol than in the long protocol (Fig. S1C), while no metabolite significantly differed between the long and short protocols after the third bout (Fig. S1F).

## DISCUSSION

### Overview of the present findings

During high-intensity exercise, ATP-producing pathways are activated to meet ATP requirements, given the small amounts of ATP readily available in skeletal muscle cells. To better understand metabolic network responses to high-intensity interval exercise, we performed metabolomic analysis using skeletal muscle samples taken from Thoroughbred horses. Here, we observed a decline in phosphocreatine content and elevations in the level of glycolytic intermediates and lactate in skeletal muscle after the first and third bouts of exercise in the long protocol, suggesting a reliance on phosphocreatine hydrolysis and glycolysis for ATP regeneration. In contrast, we found no significant changes in any metabolite concentration in skeletal muscle, albeit there was a greater reduction in the arterial oxygen saturation level across the third bout of the short protocol. These observations suggest a shift toward oxidative metabolism from substrate phosphorylation during repeated bouts of high-intensity exercise performed with short rest intervals, even though exercise intensity and duration were the same in each bout.

### High-energy phosphate metabolism

Phosphocreatine hydrolysis occurs during intense exercise or muscle contraction. In the present study, phosphocreatine content before the third bout in the long protocol, but not in the short protocol, was comparable to that before the first bout. This observation suggests that a 2 min rest interval is not sufficient to replenish muscle phosphocreatine reserves under the current exercise conditions. Additionally, phosphocreatine levels decreased to a similar extent during the first and third bouts of the long protocol. However, phosphocreatine content did not significantly decrease during the third bout of the short protocol. These observations may suggest that ATP provision via phosphocreatine hydrolysis is marginal overall during the latter bout of high-intensity exercise performed with a short rest interval.

### Glycolytic metabolism

Reactions catalyzed by glycogen phosphorylase (PHOS), hexokinase (HK), phosphofructokinase (PFK) and pyruvate kinase (PK) are considered rate-regulating reactions in glycolysis. In the present report, Thoroughbreds performed exercise at the intensity requiring maximum oxygen uptake, where glycogen is the primary substrate and extracellular glucose provides a small amount of the total (Katz et al., 1986). Therefore, it is likely that the contribution of flux through HK to overall glycolysis is minimal and that PHOS sets the potential upper limit for the rates of overall glycolysis. The present observation that the levels of the intermediates of upper glycolysis (e.g. glucose 6-phosphate and fructose 6-phosphate) increased in the first and third bouts of the long protocol may suggest increased flux through PHOS. Nevertheless, the concentrations of glycolytic intermediates between fructose 1,6-diphosphate and pyruvate did not significantly differ among the groups, despite significant differences in muscle lactate levels. This is probably because another rate-limiting enzyme, PFK, controls the conversion of fructose 6-phosphate into fructose 1,6-diphosphate and because fructose 1,6-diphosphate, an allosteric activator of PK, expedites a rate-limiting step of the PK reaction (Jurica et al., 1998), driving lactate formation without accumulating lower glycolytic intermediates.

### Regulation of glycolytic enzymes

A decrease in pH due to exercise exerts an inhibitory effect on glycolytic enzymes (Krebs et al., 1964; Trivedi and Danforth, 1966). In the current investigation, blood pH declined during the short protocol, suggesting that muscle pH also decreased progressively during the short protocol, which potentially blunts the enzymatic reactions involved in glycolysis. This could be a partial explanation for the relatively small increase in glycolytic intermediate and lactate concentrations during the third bout of the short protocol compared with the first and third bouts of the long protocol. Citrate is another potential factor that hampers the activity of glycolytic enzymes (Garland et al., 1963; Parmeggiani and Bowman, 1963). However, the increases in glycolytic intermediate and lactate concentrations were similar between the first and third bouts in the long protocol, despite an elevated citrate concentration before the start of the third bout in the long protocol. Citrate is, therefore, unlikely to be a potent inhibitor of glycolytic enzymes under the present exercise conditions.

### Lactate and glycerol 3-phosphate production

Lactate concentration primarily depends on the balance between its production and oxidation. The increase in muscle lactate levels observed in the present study indicates that the rate of pyruvate production through glycolysis exceeded the rate of oxidation through pyruvate dehydrogenase (PDH). In contrast, the lactate concentration did not significantly change during the third bout of the short protocol, representing a better match between lactate production and oxidation.

Among the detected metabolites, glycerol 3-phosphate (G3P) concentration exhibited the most significant correlation with lactate level (Fig. S2). G3P is synthesized by the reduction of dihydroxyacetone phosphate (DHAP), an intermediate in glycolysis. Similar to the conversion of pyruvate into lactate, this reaction serves to regenerate NAD^+^, which is an essential oxidant for the catalysis of glyceraldehyde 3-phosphate dehydrogenase (GAPDH) in glycolysis. Therefore, the significant increases in G3P concentration after the first and third bouts in the long protocol presumably maintain the glycolytic rate. Through the glycerol phosphate shuttle, G3P is oxidized to DHAP by coupling with the reaction of mitochondrial G3P dehydrogenase, by which the reducing equivalent is transferred to ubiquinone for subsequent oxidative ATP production. Thus, the increase in G3P concentration may also indicate the imbalance between glycolysis and oxidative metabolism.

### Acetyl-CoA and carnitine metabolism

As the capacity of PDH to produce acetyl-CoA is much greater than the flux through the TCA cycle, acetyl-CoA accumulates during high-intensity exercise (Carter et al., 1981; Constantin-Teodosiu et al., 1991). Mitochondrial carnitine acetyltransferase buffers excess acetyl-CoA to prevent it from inhibiting enzymatic flux and trapping available free CoA, by producing acetylcarnitine at the expense of free carnitine while releasing free CoA. In the current study, although acetylcarnitine levels were not evaluated, carnitine concentration decreased after the first and third bouts in the long protocol, suggesting that acetyl-CoA production through PDH exceeded the flux through the TCA cycle. In contrast, the lack of a significant decrease in carnitine content without significant acetyl-CoA accumulation in the third bout of the short protocol may suggest a better matching between flux through PDH and the TCA cycle. In addition to buffering excess acetyl-CoA, carnitine serves as an essential substrate for long-chain fatty acid transport and oxidation at the mitochondrial membrane level (Stephens et al., 2007). Accordingly, the observation of lower carnitine levels may indicate impaired fat metabolism during exercise under the present conditions.

### Mechanisms for TCAI expansion

The expansion of the TCAI pool occurs during the initial phase of exercise (Gibala et al., 1997a,b; Sahlin et al., 1990). Although various carboxylation and decarboxylation reactions regulate carbon flux into and out of the cycle, the alanine aminotransferase (AAT) reaction, in which pyruvate and glutamate are converted to 2-oxoglutarate and alanine, is quantitatively considered the most important for the increase in TCAI content at the onset of exercise (Gibala et al., 1997a). The activity of AAT is considerably high in rodent (Molé et al., 1973) and equine (Guy and Snow, 1977) skeletal muscles, and therefore the catalytic reaction of this enzyme is considered to be near equilibrium. In the present study, the changes in intramuscular alanine content showed a similar pattern to that of TCAIs, as has previously been reported in humans (Gibala et al., 1997a). Additionally, we observed that the intramuscular content of alanine and glutamate reciprocally changed before and after each exercise bout. Collectively, these findings support the notion that anaplerosis and cataplerosis of TCAI metabolites occur predominantly through the AAT reaction during exercise and recovery from exercise.

### Role of TCAI accumulation

The current understanding of anaplerosis is that the increase in TCAI content during exercise primarily represents a sink for pyruvate when its rate of formation through glycolysis exceeds its rate of oxidation in the TCA cycle (Gibala et al., 2000). This interpretation is analogous to the mass action theory proposed to explain an increase in lactate levels in skeletal muscle during exercise (Spriet et al., 2000). Supporting this view, previous studies have shown that a decline in glycogen breakdown following endurance training reduced both TCAI and lactate accumulation during exercise (Dawson et al., 2003; Howarth et al., 2004). In the current study, we observed similar changes in lactate and TCAI concentration before and after the first and third bouts in the long protocol. However, the muscle lactate concentration after the third bout in the SHORT protocol was significantly higher than that after the first and third bouts in the long protocol, despite there being no significant differences in TCAI levels among the three post-exercise groups. This dissociation between lactate and TCAI concentration probably occurred because, provided that there is sufficient pyruvate supply, the flux through the AAT reaction and thus the TCAI pool size are determined by pre-exercise glutamate availability (Bruce et al., 2001; Gibala et al., 2002).

### TCAI pool and oxidative metabolism

Previously, it had been theorized that an increase in TCAI concentration is necessary to attain high rates of oxidative energy provision. This theory was conceived from observations that TCA cycle expansion was impaired in patients with muscle phosphorylase deficiency (Wagenmakers et al., 1990) and that muscle TCAI concentrations decline with prolonged, fatiguing exercise (Sahlin et al., 1990). However, previous work has shown that manipulation of TCAI concentration does not change markers of non-oxidative energy provision (phosphocreatine, ATP and lactate) during exercise (Bruce et al., 2001) or during muscle contraction (Dawson et al., 2005). In the present investigation, we observed a greater reduction in *S*a_O_2__ levels after the third bout in the short protocol compared with the first and third bouts in the long protocol, despite comparable TCAI concentrations after each exercise bout. Although we cannot rule out the possibility that higher TCAI content at the onset of the third bout in the short protocol contributed to the greater decrease in *S*a_O_2__ level, our data may support the previous notion that the sum of TCAI content is not causally linked to the rate of mitochondrial respiration. Instead, the ability to sustain flux through anaplerotic pathways is proposed to be important for normal oxidative metabolism in muscle (Walton et al., 2003).

### Dissociation of spans I and II in the TCA cycle

It has been proposed that the TCA cycle operates in two spans: span I (from citrate to 2-oxoglutarate) and span II (from succinate to oxaloacetate) (Randle et al., 1970). In the current report, levels of span I intermediates before the third bout in the long protocol did not return to levels similar to those of the first bout pre-exercise, despite a significant decrease in the level of Σ span II metabolites. Likewise, a previous study reported that citrate concentration showed a large increase and was the only TCAI that had a higher concentration during 2 min recovery compared with that at the end of 5 min exercise, although most other individual TCAIs, except for 2-oxoglutarate, tended to have lower concentrations (Gibala et al., 1997a). Another study noted an increase in muscle citrate levels during recovery from intense cycling exercise, where inhibition of the isocitrate dehydrogenase reaction, in which isocitrate is converted to 2-oxoglutarate, was considered responsible for this phenomenon (Essén and Kaijser, 1978). However, this is unlikely to be the case for the current study, given the similar changes in the levels of 2-oxoglutarate and other span I substrates.

At the onset of the third bout of exercise in the long protocol, lactate returned to a level similar to that of the first bout pre-exercise. This observation is most likely to be the result of mitochondrial lactate oxidation because numerous studies have demonstrated that oxidation is the ultimate fate of lactate (Bergman et al., 1999; Brooks and Gaesser, 1980; Kelley et al., 2002; Mazzeo et al., 1986). During lactate oxidation, lactate is converted to acetyl-CoA, which then enters the TCA cycle by being condensed into citrate in conjunction with oxaloacetate. Oxaloacetate is provided from the remaining pool of span II substrates for citrate formation. Conversion of aspartate into oxaloacetate via the malate–aspartate shuttle can also contribute to citrate synthesis (Maizels et al., 1977), as we observed that aspartate levels were significantly lower before the third bout in the long protocol than before the first bout. The rate of the TCA cycle is determined by the flux through 2-oxoglutarate dehydrogenase (Cooney et al., 1981; Russell and Taegtmeyer, 1991; Taegtmeyer, 1983), suggesting that the flux of the TCA cycle can be stemmed at the level of 2-oxoglutarate, which then results in the accumulation of span I substrates. Taken together, it is plausible that lactate-derived carbon influx into the TCA cycle along with a decline in the flux through 2-oxoglutarate dehydrogenase during recovery from exercise results in disproportionate changes in span I and span II concentrations.

### Conclusions

To our knowledge, this is the first study to perform metabolomic analysis of skeletal muscle during high-intensity interval exercise with two distinct rest interval durations. In the first and third bouts of the long protocol, we observed a significant decrease in phosphocreatine content and significant increases in glycolytic intermediate and lactate content, suggesting a reliance on substrate phosphorylation. During the third bout of the short protocol, we found a greater reduction in *S*a_O_2__ levels, suggesting a shift toward oxidative phosphorylation during repeated bouts of exercise performed with short rest intervals. Nevertheless, there were no metabolite concentrations that significantly changed, indicating that the activity of each energy production system of the latter bout during high-intensity exercise performed with a short rest interval is not necessarily reflected in apparent changes in metabolite concentrations. This presumably results from a better matching between metabolite flux into and out of the pathway and cycle, as well as between metabolite production and disposal. The current study indicated substantial variation in metabolite concentrations depending on the number of repetitions and the duration of rest intervals between exercises, even when the exercises themselves were identical. Our findings, which use metabolomics technology to decipher the interconnection of metabolic pathways during high-intensity exercise, may contribute to future studies of the complex integrative nature of exercise-regulated molecular metabolic networks.

## Supplementary Material

10.1242/jexbio.246896_sup1 Supplementary information

Table S1. Concentrations of detected metabolites
