# Supplementary material for: Metabolomic responses to high-intensity interval exercise in equine skeletal muscle: effects of rest interval duration
Source: J Exp Biol. 2024 Feb 16;227(4):jeb246896. doi: 10.1242/jeb.246896 (PMC10911116; doi:10.1242/jeb.246896)
Supplement: Supplementary information [file jexbio-227-246896-s1.pdf]

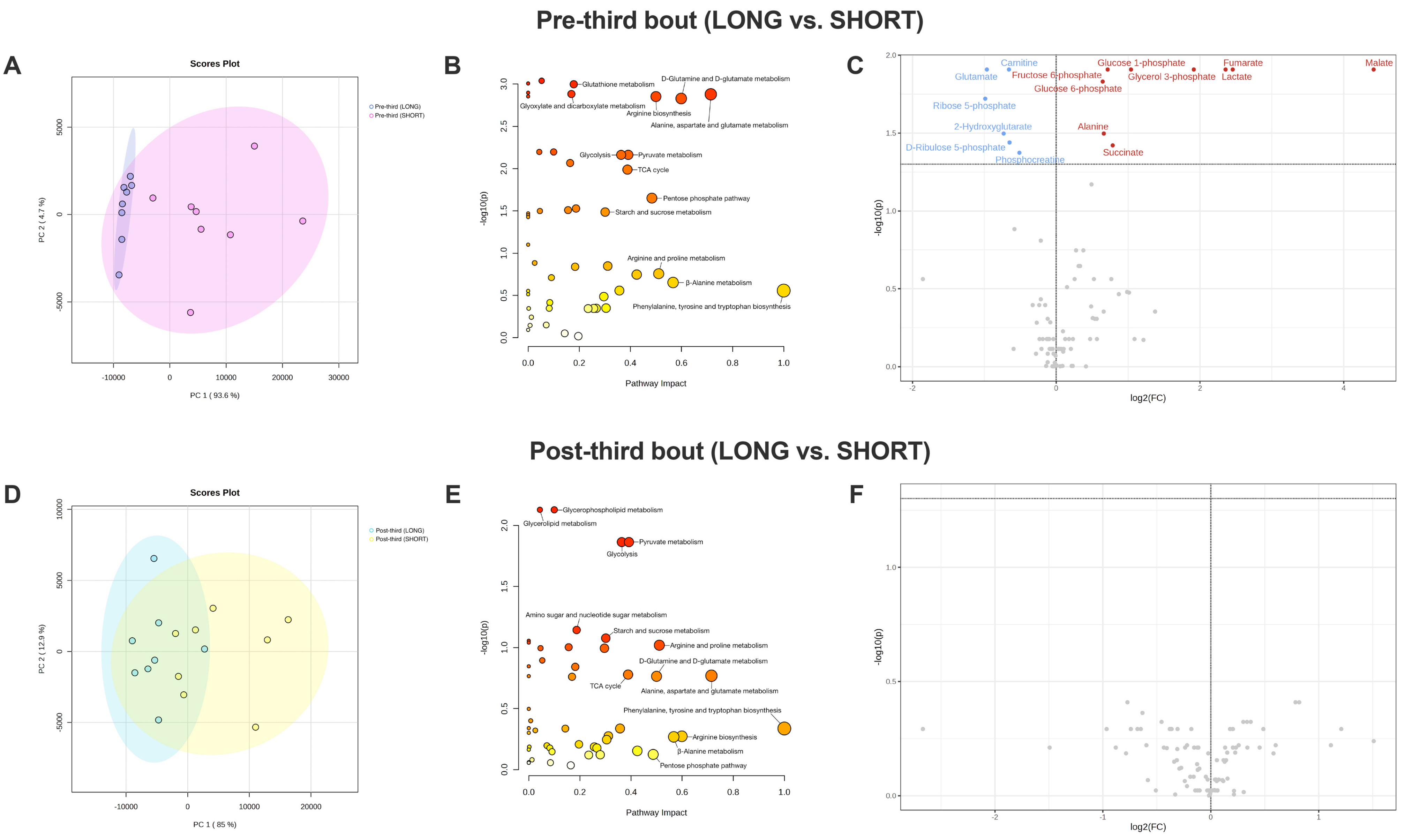

Fig. S1. Comparison between the long and short protocols at pre- and post-third exercise bout. PCA (A,D), pathway impact analysis (B,E) and volcano plot (C,F) before and after the third exercise bouts in the long and short protocols. PC1 and PC2 were plotted with their 95% confidence intervals. Pathway impact analysis was performed using the Kyoto Encyclopedia of Genes and Genomes (KEGG) database. Metabolite concentrations between the two exercise protocols were compared using paired t-tests, followed by the method of Benjamini and Hochberg (FDR<0.05).

Journal of Experimental Biology • Supplementary information

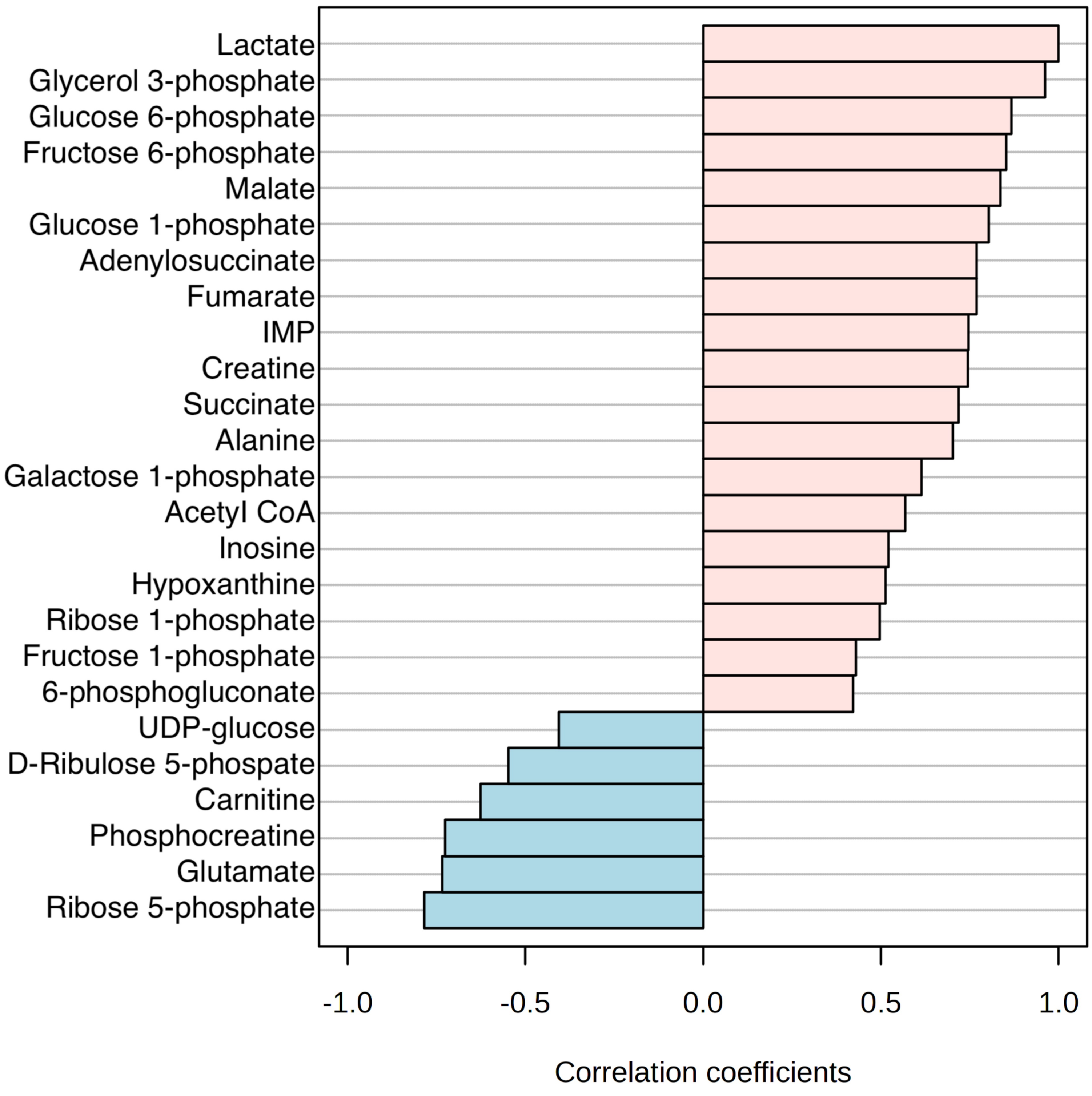

Fig. S2. Top 25 metabolites correlated with lactate. Correlation between lactate and other metabolites was analyzed using Pearson's correlation coefficient.

Table S1. Concentrations of detected metabolites

Available for download at
https://journals.biologists.com/jeb/article-lookup/doi/10.1242/jeb.246896#supplementary-data

Journal of Experimental Biology • Supplementary information
